# A Case in Practice

**Published:** 1906-08

**Authors:** Doisier Leitner

**Affiliations:** Ocala, Fla.


### A CASE IN PRACTICE.

Dr. Doisier Leitner, Ocala, Fla.

Florida State Dental Society, 1906.

The subject that I desire to present to you is: "A Case inPractice", and in presenting this paper to the society, I desire to state that I am not posing as a specialist in the treatment of pyorrhea, but realizing that we cannot put too much stress on this most important subject, I will endeavor to tell you about a most wonderful case we had and its marvelous results.

A woman, about thirty- five years old, presented herself at the chair for treatment and when we had made a thorough examination of her mouth, it looked as though a cure were impossible, for it was one of the worst kept mouths that I have ever seen; but we said that if she would do her part, we would endeavor to perfect a cure.

Upon examination we found two of her teeth, the first and second lower molars on the left side, beyond redemption and they had to be extracted. All others were loose in their sockets and could be easily shaken and of course the gums were in a terrible hypertrophied condition. She had suffered from the effects of mercurial poisoning two or three times and all of her teeth were literally covered with calculus.

Her general health was poor, her physician said she had tuberculosis and such appeared to be the case. Her breath was so very foul that her children dreaded to kiss her. She had abandoned the care of her teeth for every time she brushed them, her gums would bleed so freely, it was disagreeable to do so.

All these things being considered, we told her that if she would submit to an almost daily treatment, we would assure her of some relief and probably a cure; so we began by removing the bulk of the salivary calculous, which came off in great flakes and prescribed an antiseptic mouth-wash to be used very frequently during the day.

The next time she came we took each tooth separately and tried our best to give it a thorough cleaning, and I may say just here, that though a man be as wise as Solomon, as patient as Job, or as determined as Jerome, he can never clean a tooth at one sitting. It takes time and patience. After one has cleaned a tooth to suit himself, let it stand a day or two and see what the gum says about it. The gum is a judge that is impartial,and if there is the least bit of accumulation left on the tooth, it will not resume its normal size nor take on the natural tint. In cleaning these teeth we used the orange-wood stick with pumice, and by this method we conld reach every surface of the tooth and especially were we particular about the cervical margins, for it is here that the tooth must be perfectly smooth and kept so.

A person need not be afraid of using the pumice too much; at first it sounds harsh to the operator's ears and has a tendency to make him nervous, but when he sees the great improvment, he likes it, and the sweet strains of Mendelssohn's Wedding March are nothing to compare to the sweet music made by the stick as it goes in, around and over the teeth.

At each sitting we bathed the gums thoroughly with tincture of iodine to stimulate them to healthy granulations and this treatment we kept up for about a month, when noting such vast improvments, we directed her to come only once a week.

We continued this once-a-week treatment for a time, then we reduced it to twice a month and finally we dismissed her and when we did her gums were entirely firm and had taken on their natural pinkish appearance, her breath was almost odorless, most of her teeth were perfectly firm and her general health almost good.

Also during our treatment we had her exercise her teeth and surrounding tissues by chewing hard substances and eating things that would require much hard mastication, thus building up, as it were, a new mouth.

This has been about six months ago. She was in the office the other day and I saw with pleasure that her mouth was still improving and she said that now she was able to eat almost anything.

The health of a person depends upon the condition of his mouth and from this illustration, we conclude that the health and prosperity of our fair South-Land—Florida, rests with us dentists and what we should endeavor to teach our patients and the people of our whole state, especially in regard to their teeth is: Cleanilness is next to Godliness.

